# CCR6 is not necessary for functional effects of human CCL18 in a mouse model

**DOI:** 10.1186/1755-1536-5-2

**Published:** 2012-01-18

**Authors:** Irina G Luzina, Sergei P Atamas

**Affiliations:** 1 Department of Medicine, University of Maryland School of Medicine, Baltimore, MD 21201, USA; 2 Baltimore VA Medical Center, Baltimore, MD 21201, USA

**Keywords:** CCL18, CCR6, fibrosis, inflammation, T-lymphocytes

## Abstract

CCL18, a chemokine with no known receptor, has been implicated in several fibrotic pulmonary diseases associated with T-lymphocyte infiltration. It has been hypothesized that CCL18 may act through CCR6. Gene delivery of human CCL18 to the lungs of wild-type mice induced pulmonary infiltration of T-lymphocytes, less than 5% of which expressed CCR6. In the lungs of CCR6-deficient mice, CCL18-driven infiltration of T-lymphocytes was attenuated but not fully abrogated. It was concluded that CCR6 is not necessary for CCL18-induced changes in mice *in vivo *and that CCR6 is not the main functional receptor for CCL18 in this model.

## Correspondence

CC chemokine ligand 18 (CCL18, also termed MIP-4, PARC, AMAC-1, DC-CK-1 and SCYA18) is a chemokine that has been implicated in several fibrotic pulmonary diseases associated with T-lymphocyte infiltration [1-5]. This cytokine has no known receptor and is present in humans but not in mice [6-9], although human CCL18 is fully functionally active in mice *in vivo*, causing chemotaxis of T-lymphocytes [3-5,9]. These observations suggest that although CCL18 was lost in mice after evolutionary separation from human ancestors, the receptor for it has been preserved in both mice and humans. Identification of a functional CCL18 receptor would allow for development of therapies targeting CCL18-driven lymphocytic inflammation and fibrosis. However, major efforts of numerous laboratories for more than a decade failed to identify a CCL18 receptor. At this point, excluding CCL18 receptor candidates becomes important for narrowing the spectrum of potential cell surface molecules that may band CCL18 and mediate its effects, and thus for avoiding duplicating the efforts of various investigators.

It has been recently suggested [10] that CC chemokine receptor 6 (CCR6) may be a functional receptor for CCL18. CCR6 is known as the receptor for a different chemokine, CCL20 (also termed LARC or MIP-3α), and human CCL20 is biologically active in mice *in vivo *and on mouse cells in culture [11,12]. Therefore, we hypothesized that if CCR6 is a receptor for CCL18, the effects of human CCL18 in mice [3-5,9] may be mediated by mouse CCR6.

## Findings

**Figure 1:**
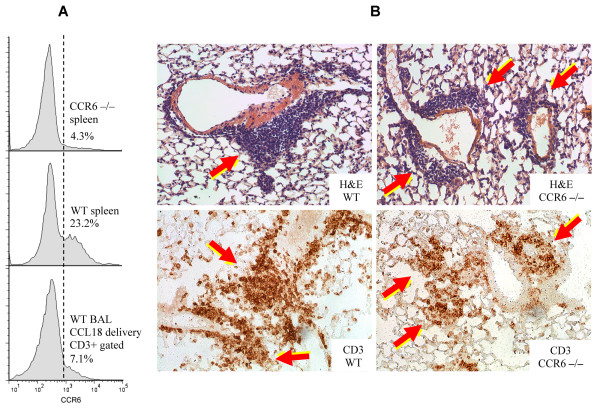
**Flow cytometric and histological analyses in CCR6-deficient and wild-type mice**. **(A) **Flow cytometry for CCR6 of unseparated spleen cells from CCR6-deficient (top) or wild-type (middle) mice, as well as bronchoalveolar lavage cells gated on CD3 from a mouse after *in vivo *CCL18 gene delivery (bottom). **(B) **Hematoxylin and eosin staining (top) and immunohistological staining for CD3 (bottom) of wild-type (left) or CCR6-deficient (right) mice.

To address this hypothesis, two types of experiments have been performed. In the first series of experiments, wild-type C57Bl/6 mice (The Jackson Laboratory, Bar Harbor, ME, USA) received intratracheal instillations of a replication-deficient recombinant adenoviral construct encoding human CCL18 (AdV-CCL18), exactly as described previously [3-5]. Control mice received similar amounts of AdV-NULL, which does not encode a cytokine. Three animals per group were analyzed in two independent experiments, with similar results. Fourteen days after instillations, bronchoalveolar lavage (BAL) was analyzed by flow cytometry, revealing a significant accumulation of lymphocytes (22.1 ± 3.5% of total BAL cells), in contrast to the number of lymphocytes found in the BAL of AdV-NULL-treated mice (2.7 ± 0.8% of total BAL cells). BAL cells were stained and analyzed with flow cytometry for CD3 and CCR6. Only a small fraction of T-lymphocytes stained positive for CCR6, whereas nearly a quarter of the splenocytes obtained from these mice were CCR6-positive (Figure 1A). These observations suggested that human CCL18 attracts mostly CCR6-negative T-lymphocytes to mouse lungs, and that mouse CCR6 is not necessary for the functional response to human CCL18 in mice.

To further address this issue, we utilized homozygous CCR6-deficient (CCR6-/-) mice (The Jackson Laboratory). The CCR6-deficient status of these animals was confirmed by flow cytometry (Figure 1A). Three CCR6-/- and three wild-type mice were instilled with AdV-CCL18 on two different occasions, and a histologic analysis of the lung tissues was performed. In all cases, gene delivery of human CCL18 caused lymphocytic infiltration of mouse lungs, as previously described [3-5], although the infiltrates appeared smaller in the CCR6-/- mice than in wild-type mice (Figure 1B). The smaller size of the infiltrates in the CCR6-/- mice may be explained by changes in the overall regulation of lymphocyte homeostasis in these animals [13]. Thus, lymphocytic infiltration of the lungs occurred in response to CCL18 in CCR6-deficient animals.

Based on these combined observations, it was concluded that mouse CCR6 is not necessary for CCL18-induced changes in mice *in vivo*, and that CCR6 is not the main functional receptor for CCL18 in this model. These findings do not exclude the possibility that mice and humans utilize different receptors for CCL18, although this is unlikely considering the remarkable similarity of CCL18 effects on human and mouse lymphocytes [3-5,9]. Further research is necessary to identify CCL18 receptor(s), as the CCL18-dependent pathway is centrally involved in a variety of maladies, particularly T-lymphocyte-mediated fibrotic pulmonary diseases [1].

## Abbreviations

AdV: adenovirus; BAL: bronchoalveolar lavage; CCL18: CC chemokine ligand 18; CCR: CC chemokine receptor; CCR-/-: homozygous CCR6-deficient.

## Competing interests

The authors declare that they have no competing interests.

## Authors' contributions

IGL participated in the experimental design, performed the experiments and participated in data interpretation. SPA conceived the study; participated in its design, coordination and data interpretation; performed statistical analyses; and wrote the manuscript. Both authors read and approved the final manuscript.
